# Prognostic value of 12 m7G methylation-related miRNA markers and their correlation with immune infiltration in breast cancer

**DOI:** 10.3389/fonc.2022.929363

**Published:** 2022-08-05

**Authors:** Wenchuan Zhang, Shuwan Zhang, Zhe Wang

**Affiliations:** Department of Pathology, Shengjing Hospital of China Medical University, Shenyang, China

**Keywords:** miRNA, RNMT, FAM103A1, m7G, immune infiltration, breast cancer

## Abstract

RNA guanine-7 methyltransferase (RNMT), in complex with FAM103A1, plays an important role in tumorigenesis and development. The aim of this study was to establish a prognostic model of RNMT and FAM103A1-based upstream microRNAs and explore its correlation with immune cell infiltration in breast cancer (BC) while investigating its potential prognostic value and verify the model by quantitative real-time polymerase chain reaction (qRT-PCR). The miRNA expression data upstream of the m7G methyltransferase complex RNMT/FAM103A1 in BC was obtained from The Cancer Genome Atlas and TargetScan databases. We performed univariate Cox regression, LASSO regression, Kaplan-Meier survival, and principal component analyses, along with risk prognostic modelling. Based on multivariate Cox regression analysis, a total of 12 m7G methyltransferase-related miRNAs were found. The model showed good accuracy for predicting the 1-, 3-,5-, and 10-year survival rates, and the areas under the curve were almost >0.7. To characterize the risk-level model constructed from 12 miRNAs, 12 differentially expressed mRNAs related to prognosis and immune infiltration were obtained. The prognosis of BC patients is well predicted by the risk model we constructed. This model is also closely related to immune infiltration, and new immunotherapy targets can be explored from this field.

## Introduction

Breast cancer (BC) is the most common cancer worldwide, surpassing lung cancer, and it has the highest incidence rate of malignancy. In 2020, there were approximately 2.3 million new cases worldwide (accounting for 11.7% of all cancer incidence rates). According to data from international research institutions, this number is expected to increase to more than 3 million by 2040 (1). However, the side effects of the traditional treatment methods (including surgery, radiotherapy, chemotherapy, and endocrine therapy) are intolerable for the patients. In recent years, immunotherapy has achieved great success in treating melanoma, non-small-cell lung cancer, acute lymphoblastic leukaemia, and other tumors. BC patients traditionally considered to have “weak immunogenicity” are expected to benefit from immunotherapy (2). Compared with traditional treatment methods, immunotherapy is well tolerated, has no toxic drug accumulation, and can prevent adverse reactions caused by systemic therapy (3). Therefore, there is an urgent need to identify novel and effective prognostic markers and therapeutic targets. The occurrence and development of tumors are closely related to genetic and epigenetic changes. Recently, m7G has been shown to play a crucial role in various stages of RNA transcription, processing, degradation, and translation. Efficient ex-pression of genes in eukaryotes requires the addition of a 7-methylguanosine cap at the 5′ end of mRNA, which is an inverted 7-methylguanosine group connected to the first transcriptional nucleotide on RNA polymerase (Pol II) transcripts (4, 5). 7-Methylguanosine is attached to the transcript through a triphosphate from the 5′ hydroxyl group to generate a structure designated as m7G (5′)PPP(5′)X (where X is the first nucleotide transcribed). This unique molecular structure within the cell is thought to specifically target the 5′ end of RNA Pol II transcripts for several gene regulatory processes, including splicing, nuclear export of mRNA, and translation initiation (6, 7). The methyl cap also protects RNA from exonucleases until it is removed by a decapping enzymes (8). Enzymes that catalyse methyl cap synthesis are essential in organisms from yeast to humans. In mammals, these enzymes are RNA guanylyltransferase, 5′-phosphatase (RNGTT), and RNA guanine-7 methyltransferase (RNMT) (9). Among them, RNMT catalyses the methylation of the cap at the N 7 position to generate a methyl cap, resulting in the m7G(5′)PPP(5′)X (10–12). Recently, a study has shown that the proliferation rate of untransformed mammary epithelial cells does not change when cellular RNMT activity is reduced by 50%, whereas some BC cell lines show reduced proliferation and increased apoptosis. While the activity of RNMT is enhanced in most BC cell lines, PIK3CA, which encodes the p110a subunit PI3Ka, is oncogenically mutated. In contrast, all cell lines insensitive to RNMT depletion expressed wild-type PIK3CA. This indicates that inhibition of RNMT activity can inhibit oncogenic mutation of PIK3CA, thereby reducing the proliferation of cancer cells (13). Studies have shown that some cellular signalling pathways can regulate the formation of mRNA caps on specific target genes, thereby regulating their expression. For example, c-Myc and E2F1 increase the phosphorylation of RNA pol II, thereby promoting mRNA cap formation by re-recruiting methyl cap synthetic enzymes (14–16). During the cell cycle, mRNA cap formation is also regulated by CDK1-dependent phosphorylation (17). However, there is no research on the role of miRNAs upstream of RNMT in regulating target genes. Some studies have found that RNMT does not function as a monomer but that it forms a CAP methyltransferase complex with FAM103A1 to promote cap maturation and maintain mRNA levels for mRNA translation and cell survival (18). Therefore, this study aimed to investigate whether there is upstream miRNA regulation of the m7G methyltransferase complex RNMT/FAM103A1. Tumor initiation and progression is a complex process that requires interactions between cancer cells, the microenvironment, and the immune system (19, 20). The importance of the microenvironment and immunomodulatory factors in BC has been known for many years (21). Recent studies have found that in the tumor microenvironment, miRNA patterns associated with the molecular signatures of BC construct a complex immune regulatory network, revealing the biological functions of miRNAs in BC extracellular matrix and immune infiltration (22). Currently, in the study of immune checkpoint inhibitors, the anti-PD-1 antibody pembrolizumab is of great significance in the treatment of triple-negative breast cancer patients. Although there are an increasing number of studies on BC immunotherapy, they are still in the preclinical or clinical trial stage (21, 23, 24). Therefore, this study aimed to investigate the existence of miRNAs upstream of the m7G-modified methyltransferase complex RNMT/FAM103A1 and to identify new prognostic markers and immunotherapy drug targets in BC.

## Results

### 12 important miRNAs are closely related to m7G methyltransferase RNMT/FAM103A1

**Figure 1:**
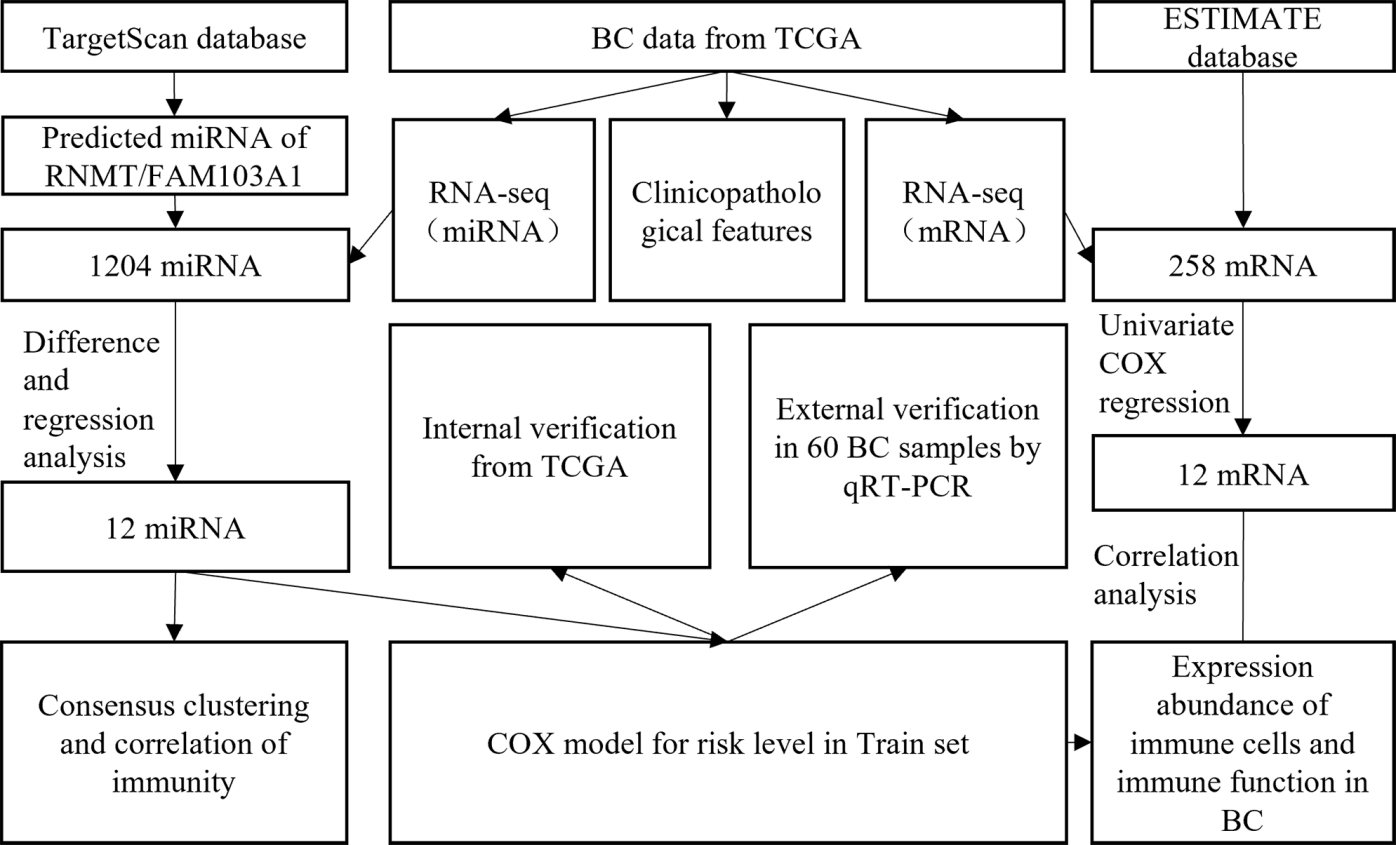
Flow chart of the present study.

**Figure 2:**
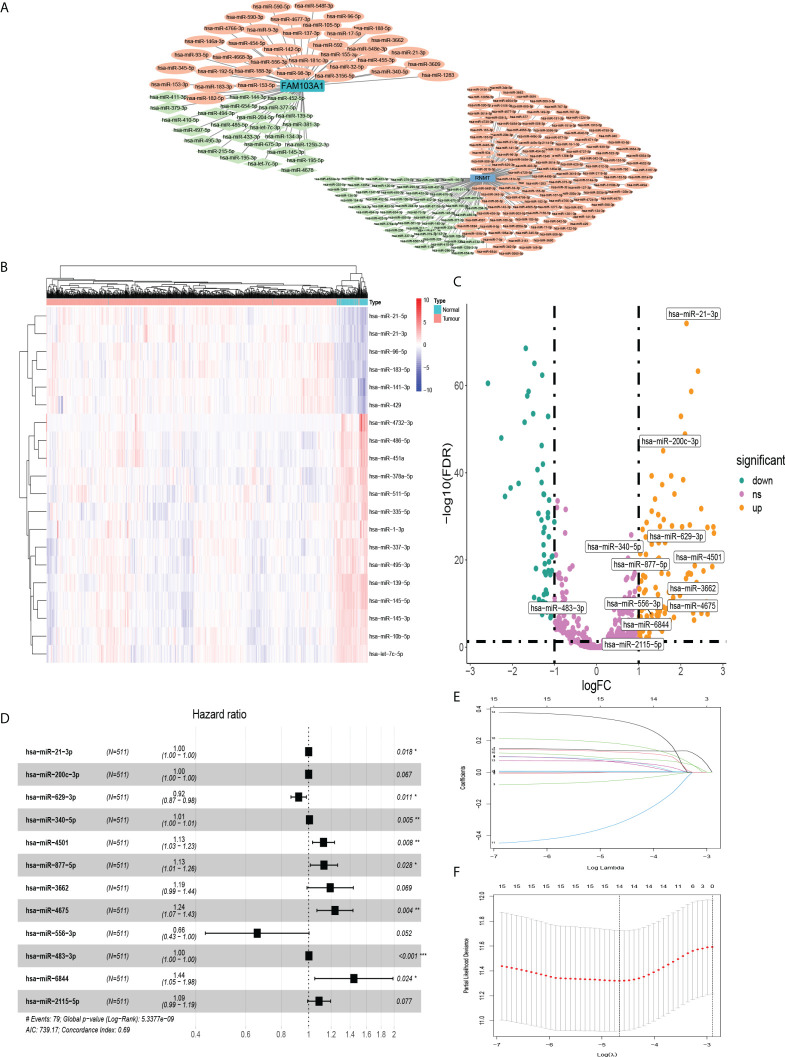
MiRNAs that were differentially expressed in BC and affected prognosis. **(A)** Co-expression network of RNMT/FAM103A1 and their upstream miRNAs. **(B)** Among the 201 upstream miRNAs of RNMT/FAM103A1 that can genetically modify by m7G, a heatmap of the top 20 most differentially expressed miRNAs in BC patients and healthy patients was drawn. Blue represents the healthy patients, and orange represents BC patients. **(C)** Volcano plot of miRNAs. **(D)** After multivariate Cox regression, a forest plot of 12 miRNAs. **(E, F)** Plots for LASSO regression coefficients. *p < 0.05, **p < 0.01, ***p < 0.001.

A research flowchart is presented in **Figure 1**. A total of 1204 predicted miRNAs related to the m7G methyltransferase target gene RNMT/FAM103A1 in BC patients were analysed for differences (**Figure 2A**), and 201 miRNAs with differences were obtained, of which 136 were upregulated differentially expressed miRNAs (logFC ≥ 1, FDR < 0.05), and 65 downregulated differentially expressed miRNAs (logFC ≤ -1, FDR < 0.05) (**Figure 2C**). A heatmap of the top 20 most differentially expressed miRNAs among the 201 miRNAs is shown (**Figure 2B**). We randomly divided the dataset into two groups according to 0.5: training group and validation group. Univariate Cox analysis was performed on 201 miRNAs, where we set the P-value to < 0.05, to obtain 16 miRNAs that have an impact on prognosis. Among these, hsa-miR-3662, hsa-miR-2115-5p, hsa-miR-483-3p, hsa-miR-21-3p, hsa-miR-6844, hsa-miR-483-5p, hsa-miR-340-5p had a more significant effect on prognosis (P<0.01) (**Supplementary Table S1**). To further obtain meaningful miRNAs for prognosis, we performed least absolute shrinkage and selection operator (LASSO) regression to screen the 14 important miRNAs (**Figures 2E, F**). These miRNAs were analyzed by multivariate regression, and finally 12 important miRNAs were obtained. Hsa-miR-21-3p, hsa-miR-340-5p, hsa-miR-4501, hsa-miR-877-5p, hsa-miR-4675, hsa-miR-483-3p, and hsa-miR-6844 were screened as independent prognostic risk factors. Hsa-miR-629-3p was independent prognostic protective factor (**Figure 2D**).

### Consensus clustering identified three clusters of BC patients with related to immunity

**Figure 3:**
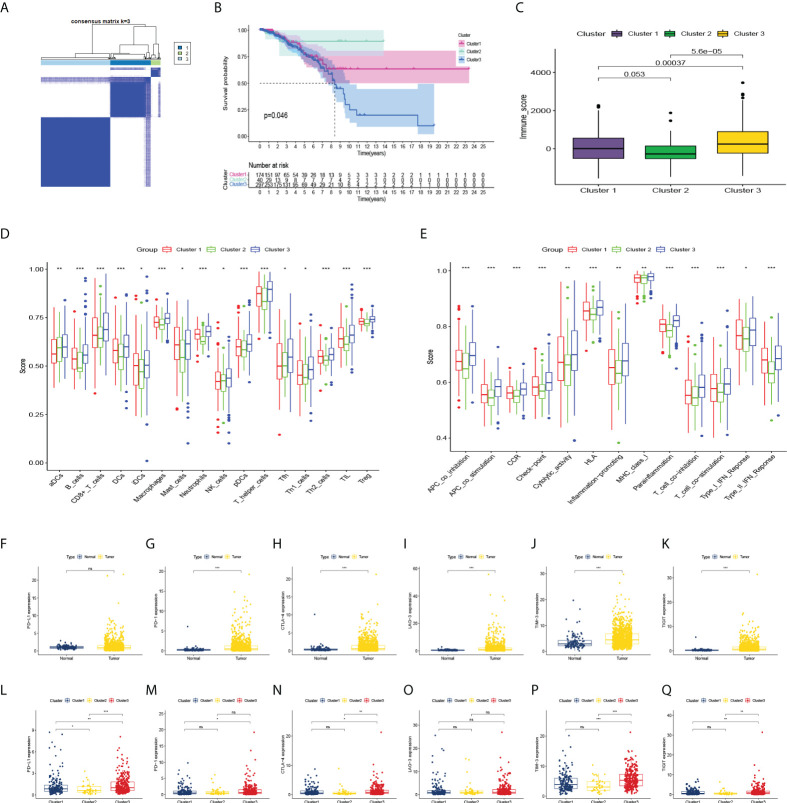
Consensus clustering and correlation of immunity. **(A)** Consensus clustering matrix for k = 3. **(B–E)** Kaplan-Meier curves, immune score, and the infiltrating levels of immune cell and immune function types of 3 clusters. **(F–K)** The expression levels of PD-L1, PD-1, CTLA-4, LAG-3, TIM-3, TIGIT in BC patients and normal people, and **(L–Q)** in 3 clusters. ns, not significant. *p < 0.05, **p < 0.01, ***p < 0.001.

Based on the miRNA data of 511 BC patients, consensus clustering was performed on gene expression profiles by the ConsensusClusterPlus package to classify tumor tissues into 3 molecular subtypes (**Figure 3A**). Among these three molecular subtypes, survival was performed. On analysis, cluster 3 had the lowest survival rate (**Figure 3B**). The three types were compared in the expression of immune cell infiltration (**Figures 3C–E**), and the expression of the current mainstream immune checkpoints was compared between groups (**Figures 3L–Q**). Except that there is no difference in PD-L1 between BC patients and normal people, others were different and the difference in expression of PD-1, CTLA-4, TIM-3, and TIGIT were more significant in cluster three patients (**Figures 3F–K**).

### Prognostic prediction ability and internal validation of the model constructed by 12 miRNAs

**Figure 4:**
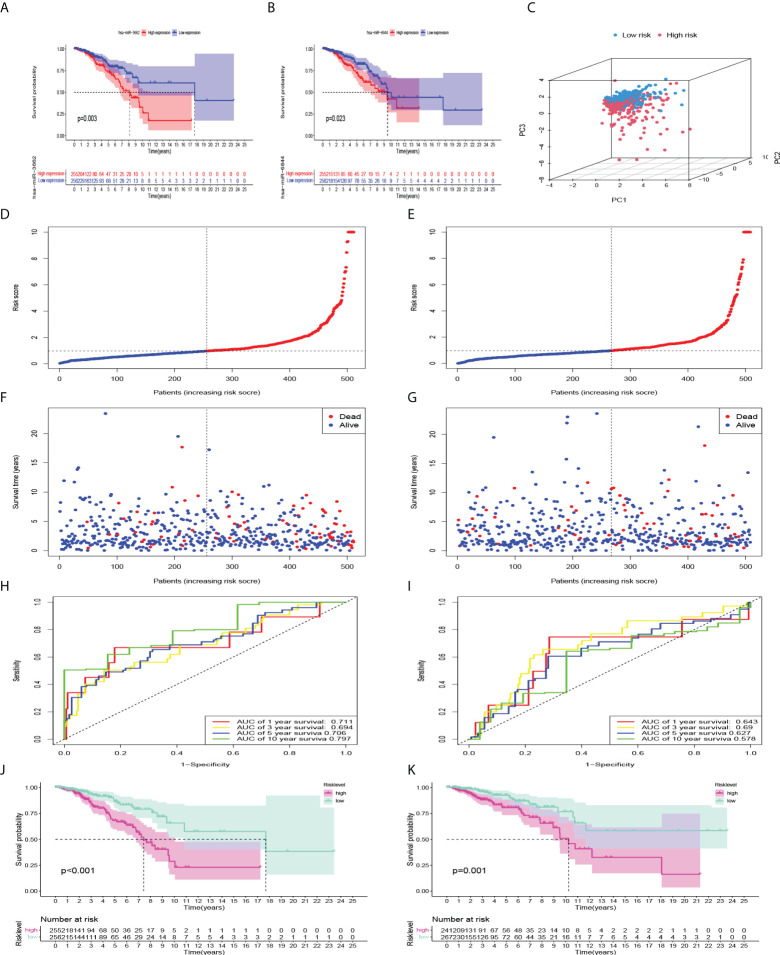
Internal verification and predictive efficiency of the Cox model consisting of 12 miRNAs. **(A, B)** Kaplan-Meier curves showed that high expression of miR-3662 and miR-6844 were significantly correlated with a poor prognosis. **(C)** PCA of the model. **(D, F)** Risk factor association map was made for each BC patient in train set, and **(E, G)** in validation set. **(H, J)** ROC curve and Kaplan-Meier curves of the model in train set, and **(I, K)** in validation set.

Kaplan-Meier univariate survival analysis was performed on the 12 miRNAs to study the effect of each factor on survival time. There was a significant correlation with patient outcomes (**Figures 4A, B**). The Cox model was constructed with these 12 miRNAs, which were divided into high- and low-risk groups. The calculating formula of risk score is:


$$Risk\;score=\sum_{i=1}^{\mathrm{12}}\;Coef_{i}*x_{i},$$

i = {1, 2, 3,…12}. *Coef_i_* means the coefficients of miRNAs in risk level model, *x_i_* is the expression values of the miRNAs. First, principal component analysis (PCA) showed that the repeatability within the group was relatively good, the sample data were very similar, and there was a good difference between the groups (**Figure 4C**). Subsequently, to evaluate the ability of the model to predict prognosis, a receiver operating characteristic (ROC) curve was constructed. The results showed that the area under the curves (AUCs) of the 1-, 3-, 5-, and 10-year survival rates were 0.711, 0.694, 0.706, and 0.797, respectively. AUC > 0.7 indicated that the model had good accuracy in predicting 1-year, 5-year, and 10-year survival rates (**Figure 4H**). Kaplan-Meier survival analysis showed that the prognosis of the high-risk group was significantly worse than that of the low-risk group (P<0.001) (**Figure 4J**). According to the risk factor association diagram, the predicted risk value for each patient is presented in ascending order. The two groups were distinguished by the median risk value: the low-risk group (blue) and high-risk group (red) (**Figures 4D, E**). The relationship between patients sorted by predicted risk value and survival time showed that the survival time of low-risk groups was slightly longer than that of high-risk groups. Among them, blue dots represent living patients and red dots represent dead patients. The number of deaths in the high-risk group was significantly higher than that in the low-risk group (**Figures 4F, G**). In the validation set, the results showed that the AUCs of the 1-, 3-, 5, and 10-year survival rates were 0.643, 0.690, 0.627 and 0.578, respectively. Kaplan-Meier survival analysis also showed that the prognosis of the high-risk group was significantly worse (P=0.001) (**Figures 4I, K**).

### External verification of the model with 12 miRNAs in BC by qRT-PCR

**Figure 5:**
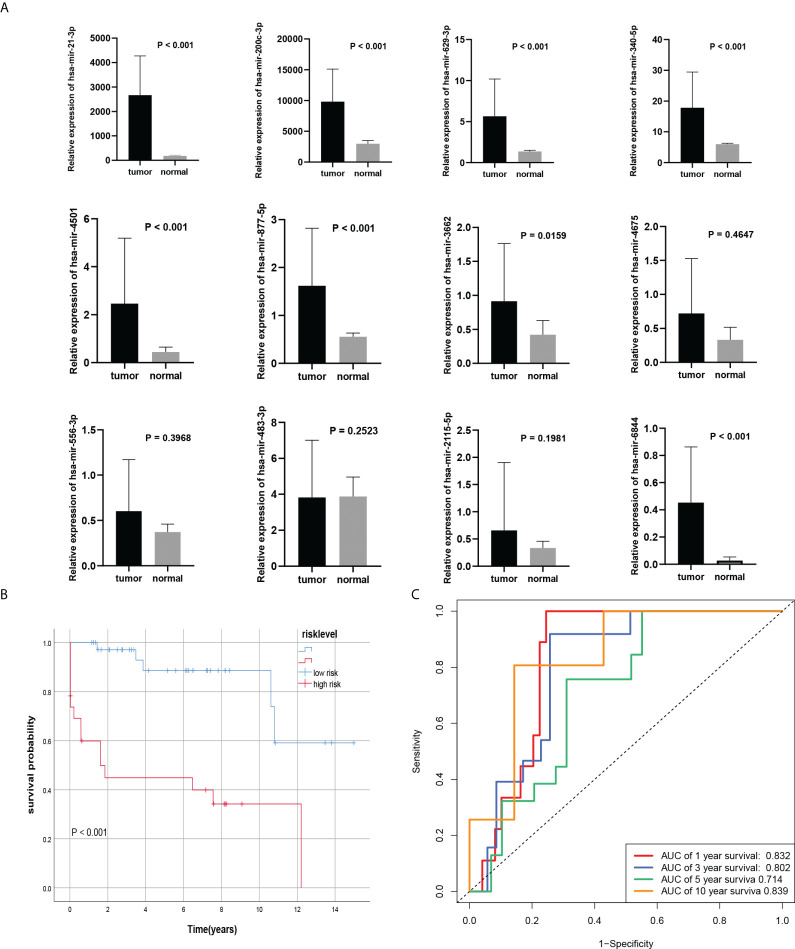
External verification of the Cox model. **(A)** 8 miRNAs were differentially expressed in BC tissues and normal tissues. **(B, C)** Kaplan-Meier curves and ROC curve of risk level model from own databases.

To verify the model we constructed, we used qRT-PCR to evaluate the expression of 12 miRNAs. We found that the expression levels of 8 miRNA in BC tissues were significantly higher than that in normal tissues. However, there was no significant difference in has-miR-4675, has-miR-556-3p, has-miR-483-3p, and has-miR-2115-5p in BC tissues compared with normal tissues (**Figure 5A**). According to the formula of the COX model, with 0.975271 as the threshold, among the 60 samples, 23 were in the high-risk group and 37 were in the low-risk group. The AUCs of the model with 12 miRNAs expressions of qRT-PCR in 1-, 3-, 5-, and 10-year survival rates were 0.832, 0.802, 0.714, 0.839, respectively (**Figure 5C**). Kaplan-Meier survival analysis showed that the prognosis of high-risk group was significantly worse than the low-risk group (P<0.001), consistent with the results of the training set (**Figure 5B**).

### Construction of risk-level model and clinicopathological features

**Figure 6:**
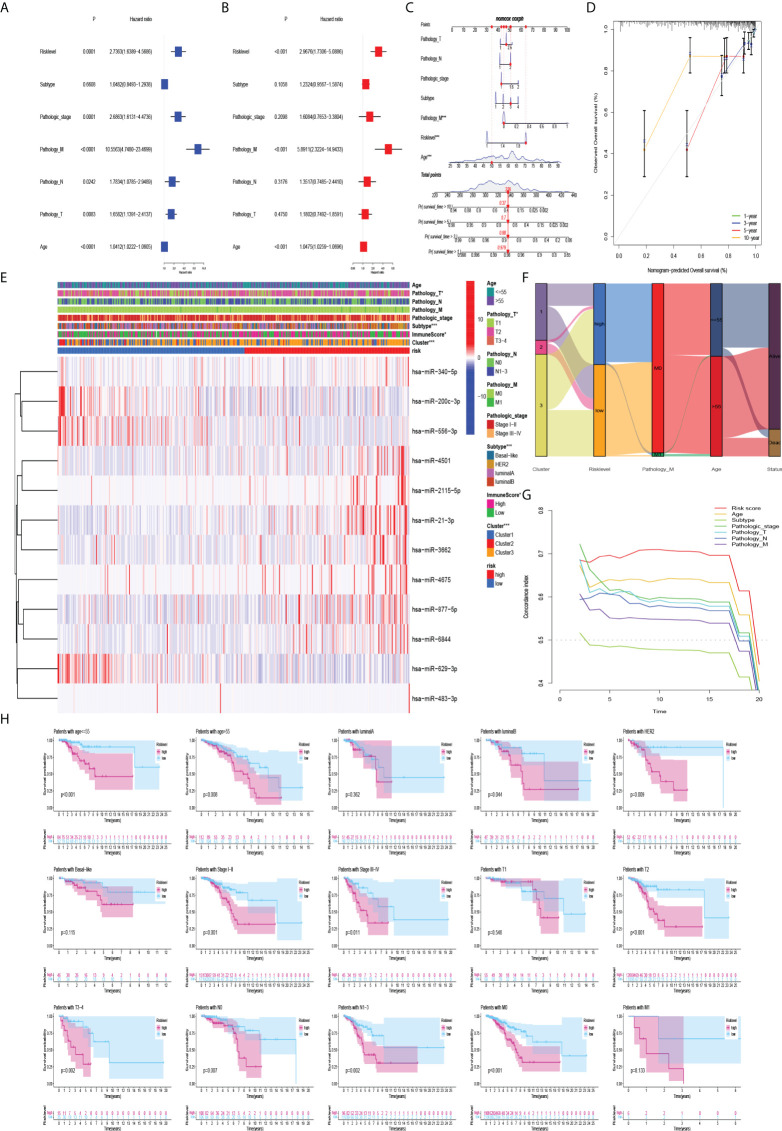
Association of model and clinicopathological features. **(A)** Univariate Cox regression analysis and **(B)** multivariate Cox regression analysis showed that risk level was an independent prognostic risk factor. **(C)** Nomogram of risk level and clinicopathological features for one of the patients. **(D)** Plot of the prediction model calibration curve. **(E–G)** Heatmaps, sankey diagram, and C-index for model and clinicopathological features. **(H)** Kaplan-Meier curves of the risk model in clinical groups.

To evaluate the predictive efficacy of the risk-level model in actual clinical practice, we combined the risk level with clinicopathological characteristics (including age, subtype, pathologic stage, and TNM stage). Univariate and multivariate Cox regression analyses revealed that risk level was an independent prognostic risk factor (P<0.001) (**Figures 6A, B**). The nomogram of the model was drawn and analysed for patient No. 1 (**Figure 6C**). Sankey diagram and heatmaps for model and clinicopathological features were also drawn. Patients with cluster 3 were more in the high-risk group (**Figures 6E, F**). To further evaluate whether the prediction model was in line with the actual situation, a calibration curve of the prediction model was drawn. The abscissa of the graph represents the prediction probability, and the prediction model predicts the possibility of event occurrence. The vertical axis represents the actual probability of the actual event rate of the patients. The green line is the fitted line for predicting 1-year overall survival, the blue line for predicting 3-year overall survival, the red line for predicting 5-year overall survival, the orange line for predicting 10-year overall survival and the grey line is the reference line. The 1-, 3-, and 5-year three fitted lines almost completely coincide with the reference line, indicating that the predictive model has a high predictive efficacy. However, 10-year survival predictions suggested an underestimation of patient survival (**Figure 6D**). Importantly, the C-index indicates that the risk score was a very accurate indicator of the predictive ability of the model (**Figure 6G**). Therefore, we validated the risk model in clinical groups. The risk model was able to accurately estimate the survival rate of patients in age groups, luminal B and HER2 subtypes, early and late stages of tumor, and whether lymph nodes metastasis (**Figure 6H**).

### Correlation between risk-level models and immune infiltration

**Figure 7:**
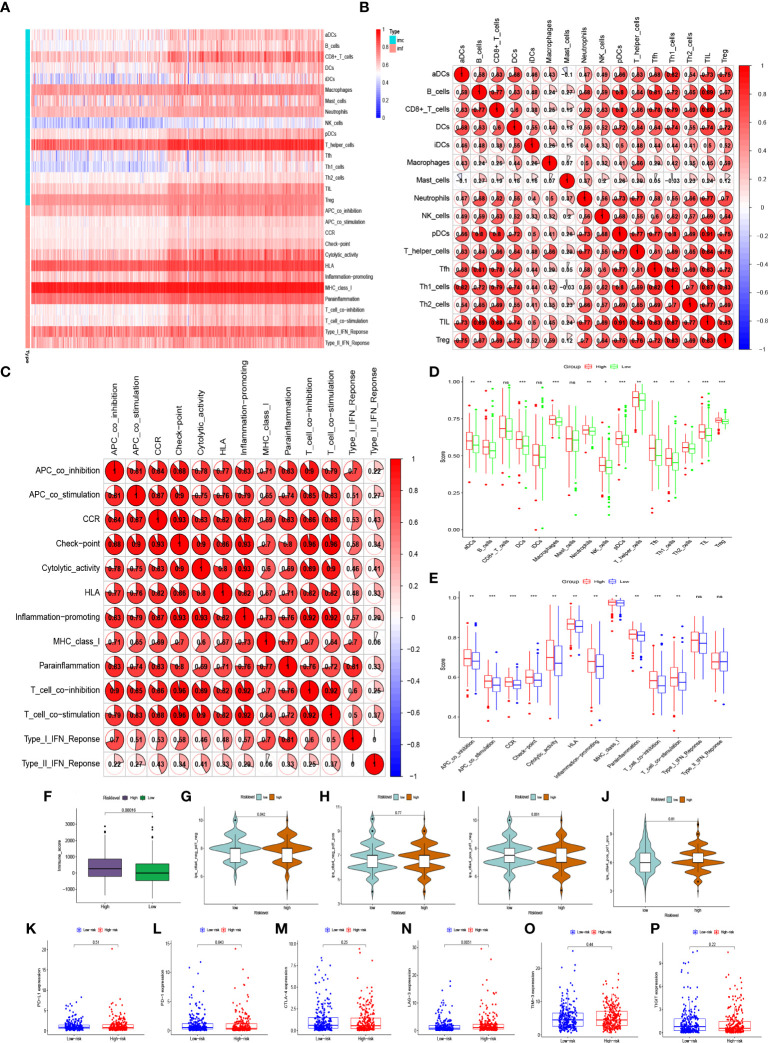
Abundant expressions of immune infiltration in BC patients. **(A)** Heatmap of expression abundances of 16 immune cells and 13 immune functions in BC patients. **(B)** Correlations between immune cells and **(C)** correlation between immune functions in BC patients. **(D)** Differences in immune cell abundance expression and **(E)** differences in abundance expression of immune function between high-risk and low-risk groups in BC patients. **(F, G–J)** Immune score and immunophenoscore of risk level groups. **(K–P)** The expression levels of PD-L1, PD-1, CTLA-4, LAG-3, TIM-3, TIGIT in high-risk and low-risk groups. ns, not significant. *p < 0.05, **p < 0.01, ***p < 0.001.

The single-sample gene set enrichment analysis (ssGSEA) algorithm was used to analyze the abundance of genes expressed by each immune cell and immune function signature in patients with BC. The heatmap of immune infiltration in BC patients revealed that the expression of T helper cells and major histocompatibility complex class 1 (MHC class I) was prominent in the tumor immune microenvironment (**Figure 7A**). Tumor-infiltrating lymphocytes (TILs) and plasmacytoid dendritic cells (pDCs) had the highest correlation in the BC immune microenvironment with an R value of 0.91; mast cells and activated dendritic cells (aDCs) were negatively correlated with an R value of -0.1 (**Figure 7B**). Immune checkpoints and T-cell co-stimulatory and co-inhibitory pathways showed the highest positive correlation with an R value of 0.96 (**Figure 7C**). In addition, immune infiltration was more pronounced in the high-risk group (**Figure 7F**). Interestingly, the expression of aDCs, B cells, dendritic cells (DCs), macrophages, neutrophils, NK cells, pDCs, T helper cells, follicular helper T cells (Tfh), helper T cells 1 (Th1 cells), helper T cells 2 (Th2 cells), TILs, regulatory T cells(Treg), APC co-stimulatory and co-inhibitory pathways, chemokine receptors (CCR), immune checkpoints, cytolytic activity, human leukocyte antigen (HLA), inflammation promotion, MHC class I, T cell co-stimulatory and co-inhibitory pathways, and parainflammation were significantly lower in the low-risk group (P<0.05) (**Figure 7D, E**). It has been suggested that BC patients with high-risk level may be candidates for immunotherapy. Interestingly, patients in the high-risk group may be more suitable for anti-LAG-3 immunotherapy (**Figures 7K–P**). Although PD-1 expression was higher in the low-risk group, patients in the low-risk group may not benefit more than those in the high-risk group because there was no difference in the immunotherapy score analysis (**Figures 7G–J, L**).

### mRNAs associated with immune infiltration in risk-level models

**Figure 8:**
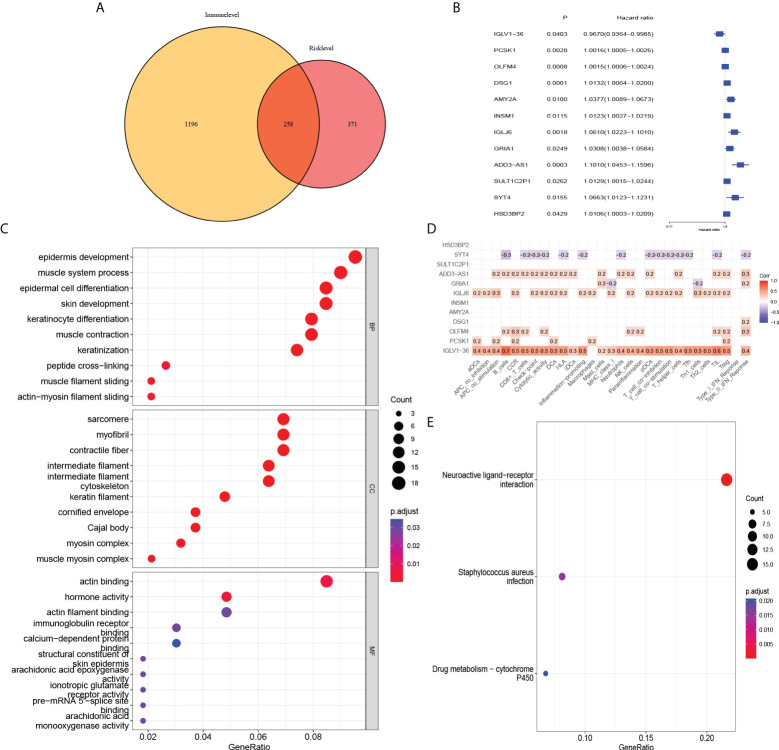
Association of risk level groups and mRNAs. **(A)** 258 mRNAs associated with risk level and immune infiltration were obtained. Subsequently, **(C)** GO enrichment analysis and **(E)** KEGG pathway enrichment analysis was performed. **(B)** By univariate Cox regression, 12 mRNAs were found to have effects on prognosis. **(D)** Correlation analysis of 12 mRNAs and immune infiltration.

To further analyse the factors affecting the risk level at the mRNA level, we found 629 differentially expressed mRNAs in the high-risk and low-risk groups in the risk-level model. The 1454 differentially expressed mRNAs from the immune score groups of the ESTIMATE database (with the median as the cut-off value) were intersected with 629 differentially expressed mRNAs to yield 258 mRNAs associated with risk level and immune infiltration (**Figure 8A**). They were enriched in epidermis development, sarcomere, actin binding, and neuroactive ligand-receptor interaction signalling pathways (**Figures 8C, E**). Univariate Cox regression analysis revealed that the 12 mRNAs were associated with prognosis (**Figure 8B**). ADD3-AS1, IGLJ6, OLFM4, PCSK1, IGLV1-36 positively correlated with immune infiltration. SYT4 genes was negatively associated with immune infiltration and correlated with poor prognosis (**Figure 8D**).

## Discussion

N 7-methylguanosine (m7G) is an essential modification of the positively charged 5′ end of mRNA in mammals that regulates mRNA export, translation, and splicing (25). Abnormal m7G modifications are closely related to the occurrence and development of various cancers (26–30). RNMT was identified as a methyltransferase that installs a subset of m7G within mRNA and affects its translational capacity (30). In addition, FAM103A1 consists of an N-terminal RNMT activating domain and a C-terminal RNA-binding domain, which functions in the m7G methyltransferase complex with RNMT (18). It is now generally accepted that miRNAs play an important role in the occurrence and development of tumors, especially in epigenetic regulation, protein interactions, and RNA metabolism (31, 32). miRNAs are a group of highly conserved, single-stranded, short non-coding RNAs. They are a key regulator of mRNA expression in both normal and abnormal biological processes, including cancer (33). Dysregulated miRNA expression has also been implicated in cell survival and proliferation as well as in cell extravasation and metastasis (34). In BC, miRNAs represent an emerging group of molecules that play critical roles in disease development and are potential tools for improving treatment and impact diagnosis (35). Therapeutic strategies based on modulating the expression levels of miRNAs and identifying their targets are promising approaches for miRNA-based molecular therapy for BC (36). Detection of circulating miRNAs has also facilitated the formation of miRNA profiles in the blood of patients with BC, emphasizing that miRNAs are promising biomarkers for early disease screening, therapeutic targets, and prediction of prognosis (37). For example, miR-21 is associated with clinical stage, lymph node metastasis, and poor prognosis (38). High miR-21 expression is also associated with poor prognosis in Asian patients with BC (39). However, it remains unclear whether miRNA regulation exists upstream of the m7G methyltransferase complex RMNT/FAM103A1.

In our study, we obtained 12 important upstream miRNAs of m7G genes. Consensus clustering classified BC patients into 3 clusters. Patients with cluster 3 may benefit more from anti-PD-1, CTLA4, TIM-3, and TIGIT immunotherapy because patients with cluster 3 had higher expression of immune checkpoints compared to the other two groups. Subsequently, we found in the heatmap and Sankey diagram that patients with cluster 3 were mainly in the high-risk group. The high-risk group, like cluster 3, had higher expression of immune infiltrates. However, patients in the high-risk group benefited more from anti-LAG-3 immunotherapy. Which grouping method is more beneficial to BC patients still needs more comprehensive evaluation and further exploration. But to a certain extent, it can be shown that these 12 miRNAs have a certain hinting effect on immunotherapy. Kaplan–Meier survival analysis found that has-miR-3662 and has-miR-6844 were all highly expressed, suggesting a worse survival rate. Kaplan–Meier analysis also revealed a difference in survival probability between the high-risk and low-risk groups. The survival rate in the high-risk group was significantly lower than that in the low-risk group. In addition, the ROC curve showed that the AUC of the model was almost >0.7, which indicates more accurate prediction of prognosis. According to the risk factor association map, the number of deaths in the high-risk group was significantly higher than that in the low-risk group. Through PCA, the model can better distinguish between high-risk and low-risk groups. Therefore, this model could serve as a potential prognostic biomarker for BC. Li et al. found that the relative expression of miR-3662 in serum exosomes was significantly higher in BC patients than healthy controls, which was shown to be valuable biomarkers to monitor patient condition in the course of surgery and chemotherapy (40). The data showed that miR-21-3p overexpression in BC was a hallmark of worse BC progression and it affected genes in pathways that drive breast cancer by down-regulating tumor suppressor genes (41). Curtaz et al. found the expression level of miR-340-5p was significantly correlated with the percentage of actively proliferating tumor cells (42). Elango et al. found, compared with patients with primary BC, the expression of miR-200-3p was decreased in BC patients with lymph node metastasis. This indicated that overexpression of hsa-miR-200-3p may inhibit BC progression and metastasis (43).In addition, eight miRNAs, miR-2115-5p, miR-483-3p, miR-6844, miR-4675, miR-877-5p, miR-4501, miR-629-3p, and miR-556-3p, have not yet been reported in PubMed for BC-related studies. Therefore, research in this area needs to be urgently conducted.

Furthermore, combining the risk level with clinicopathological characteristics (age, subtype, pathologic stage, TNM stage), we found that the risk level had a significant impact on prognosis (P< 0.001). The calibration curve showed that the predicted survival rates of the model at 1, 3, and 5 years were in good agreement with the actual situation. Interestingly, we found that the risk model had a more pronounced effect on prognosis in BC patients with HER2 subtype, so we collected paraffin samples from BC patients with HER2 subtype in our hospital for external validation. Interestingly, except that has-miR-4675, has-miR-556-3p, has-miR-483-3p, and has-miR-2115-5p were not statistically different between BC tissues and normal tissues, the expression of the other 8 miRNAs in BC tissues was significantly higher than that in normal tissues. In BC patients with HER2 subtype, patients in the high-risk group had a significantly worse prognosis than these in the low-risk group. The AUC of the model also indicated that the model had good accuracy in predicting 1-year, 5-year, and 10-year survival rates. Due to population differences, we found no differences in the expression of 4 miRNAs. In addition, because of the confounding of other molecular subtypes in the TCGA database, the power of the model to predict prognostic accuracy is lower than in our own database. And we found that the model was also very reliable in the internal validation set. Previous studies have found that miRNAs in the immune system play key roles in the developmental fate of lymphocytes and in innate and adaptive immunity (44, 45). Abnormal expression of certain miRNAs in BC may be related to immune system dysfunction. For example, the BC-derived exosomal lncRNA SNHG16 can promote the expression of miR16-5p by targeting the TGF-β1/SMAD5 pathway, thereby inducing the differentiation of CD73+ γδ1 Treg cells (46). The elevated expression level of miR-182 in the tumor tissues of BC patients may exert an immunosuppressive effect by inducing Treg cell differentiation (47). Therefore, to explore whether miRNAs upstream of the m7G gene in the model are associated with immune infiltration in the BC tumor microenvironment, we first analyzed the expression abundance of immune cells and immune functions in BC patients. In addition to iDCs, NK cells, and Th1 cells with low expression, other immune cells and immune functions, especially T helper cells and MHC class I, are highly expressed in BC. Li et al. found that blocking TGF-β signalling in CD4+ Th cells can trigger vasculature reorganization, leading to tumor hypoxia and BC cell death. Thus, blocking TGF-β signalling in T helper cells could elicit an effective cancer defense response, thus offering the potential for BC immunotherapy (48). Therefore, in our BC samples with high expression of 11 miRNAs, reducing miRNA expression in BC patients may benefit not only targeted therapy drugs, but also immunotherapy. Furthermore, we analyzed the differences in BC immune infiltration between the high- and low-risk groups. Surprisingly, except for CD8+ T cells, iDCs, Mast cells, type I interferon response, and type II interferon response, other expressions were significantly higher in the high-risk group. Kaplan- Meier analysis of the model indicated that high immune infiltration in the high-risk group was associated with poor prognosis. This further may indicate that the high-risk group is more suitable for immunotherapy than low-risk groups, especially in HER2 BC subtypes. Therefore, the 12 important miRNAs identified in this study may provide new targets for BC immunotherapy.

To further characterize the risk-level model, we subsequently obtained 258 mRNAs based on the differences between the high and low immune component groups in the ESTIMATE database of BC patients. As with our immune-related model results, mRNAs representing these models were analyzed using Gene Ontology (GO) and Kyoto Encyclopedia of Genes and Genomes (KEGG) pathway enrichment analyses. These genes were enriched in epidermis development, sarcomere, actin binding, and neuroactive ligand-receptor interaction signalling pathways. Among the 12 mRNAs associated with prognosis, IGLV1-36 was positively correlated with immune infiltration and were associated with good prognosis. This suggest that this gene may be an immune-related gene and its high expression may inhibit tumor progression in BC patients. Existing studies report that IGLV1-36 is suitable for confirming the diagnosis of POEMS syndrome (49). While its role in BC has not been reported, this may be another research target for immune infiltration. However, further verification and in-depth research are needed to determine whether these markers can become targets for new immunotherapy and the related methylation mechanisms.

## Materials and methods

### Breast cancer data and acquisition of upstream miRNA of m7G methyltransferase RNMT/FAM103A1

We obtained transcriptome mRNA-seq, miRNA-seq gene expression data, and corresponding clinical information of BC patients from TCGA database (https://portal.gdc.cancer.gov/), including 1019 tumor samples and 103 healthy samples, and downloaded human miRNA target gene files from the TargetScan database (50). Then, using Perl software, we obtained mRNA, miRNA gene expression matrix, and clinical data of BC patients according to age, subtype, pathologic stage, TMN stage, survival time, and survival status. Using R language software, 2638 miRNAs of target genes RNMT and FAM103A1 were obtained and intersected with 2217 miRNAs co-expressed in BC patients and healthy patients to obtain 1204 upstream miRNAs of m7G methyltransferase target gene RNMT/FAM103A1 in BC patients and healthy persons.

### Construction of a risk-level model for m7G-related miRNAs

Using the R software package (Limma package and edgeR package), 1204 miRNAs of m7G methyltransferase target gene RNMT/FAM103A1 in BC patients (n = 1019) and healthy controls (n = 103) were analyzed with logFC ≥ |1|, FDR < 0.05 for differential expression analysis. We randomly divided the dataset into two groups according to 0.5: training set and validation set. Then, the survival package was used to conduct univariate Cox regression analysis on 201 miRNAs, where the P value is set below 0.05, and 16 miRNAs related to prognosis were obtained. To further exclude unimportant variables and obtain less meaningful variables, we used the glmnet package to perform least absolute shrinkage and selection operator (LASSO) regression to screen out 14 important miRNAs and then carried out multivariate analysis and finally constructed a Cox model with 12 miRNAs. The high- and low-risk groups were divided into two groups. Consensus clustering identified three clusters of BC patients and explored the correlation between cluster and immunity. The Rtsne package was used to perform PCA analysis, the survival package to draw the Kaplan-Meier survival curve, and the timeROC package to evaluate the ability of the model to predict prognosis. In addition, we constructed a risk factor association map.

### miRNA extraction and quantitative real-time polymerase chain reaction

We totally collected 60 BC samples from patients and 30 normal breast tissues who underwent surgical treatments in Shengjing Hospital of China Medical University from 2007 to 2021. In these 60 BC samples, they were HR-, HER2+++ on immunohistochemistry or HR-, HER2++ on immunohistochemistry with fluorescence *in situ* hybridization (FISH) indicating that HER2 was amplified. Formalin fixation and paraffin embedding (FFPE) were to preserve the specimens. The study was approved by the hospital institutional ethics review committee. For evaluating the expression levels of 12 miRNA, we deparaffinized these specimens using xylene and ethanol. According to the manufacturer’s protocol, we extracted total RNA (including miRNAs) from FFPE tissue samples using TRIzol (Thermo Fisher Scientific, US), and cDNA synthesis was carrying out by using Mir-X miRNA qRT-PCR SYBR Kits (Takara Bio Inc., Kusatsu, Japan). Then, we performed real-time PCR reaction using One Step TB Green^®^ PrimeScript™ RT-PCR Kit (Perfect Real Time) (Takara Bio Inc., Kusatsu, Japan) on The LightCycler 480 Real-Time PCR System. 12 miRNAs expression levels were calculated by the 2^-ΔΔCt^ method and the cycle threshold (CT) values of miRNAs were normalized to the level of U6 as internal reference. Primers sequences used in our study were shown in table (**Supplementary Table S2**).

### Nomogram of risk level and clinicopathological features

The Cox model with risk level and clinicopathological characteristics (including age, subtype, pathologic stage, and TNM stage) was built using the survival package of the R language software. Through univariate and multivariate Cox regression, the impact of risk level on prognosis in clinical practice was evaluated. We then used the rms package to draw a nomogram for the Cox model, selected the fourth BC patient in the file to draw a nomogram, and used the calibration function to draw a calibration curve.

### Correlation of differentially expressed mRNAs with immune infiltration in risk-level models

Differential analysis between the high-risk and low-risk groups of BC patients was performed using R software packages (Limma package and edgeR package). In total, 629 differentially expressed mRNAs were identified. Immune score data of BC patients were downloaded from the ESTIMATE database (https://bioinformatics.mdanderson.org/estimate/), which was divided into high and low immune component groups with the median as the cut-off. A total of 1454 differentially expressed mRNAs in the two immune component groups were intersected with the differentially expressed mRNAs in the risk level groups to obtain 258 differentially expressed mRNAs associated with immune infiltration and risk level in BC patients (logFC ≥ |1|, FDR < 0.05). Gene Ontology (GO) and Kyoto Encyclopedia of Genes and Genomes (KEGG) pathway enrichment analyses were performed on 258 differentially expressed mRNAs. Using univariate Cox regression, we obtained 12 differentially expressed mRNAs that were associated with immune infiltration and risk levels in patients with BC which had an impact on prognosis. The ssGSEA algorithm in the GSVA package was used to calculate the abundance of genes expressed by each immune cell and immune function in BC patients and to draw a heat map and conduct correlation analysis between immune cells and immune functions. The expression of immune cells and immune function were explored in the high-risk and low-risk groups. Finally, we explored the association of immune cells and immune function with 12 differentially expressed mRNAs that were associated with immune infiltration and risk level in patients with BC and had an impact on prognosis.

## Data availability statement

The original contributions presented in the study are included in the article/**Supplementary Material**. Further inquiries can be directed to the corresponding author.

## Ethics statement

The studies involving human participants were reviewed and approved by the institutional ethics review committee of Shengjing Hospital of China Medical University. Written informed consent for participation was not required for this study in accordance with the national legislation and the institutional requirements.

## Author contributions

All authors contributed to the work presented in this paper. Conceptualization, WZ and ZW; TCGA resources, visualization, and analysis, SZ; writing—original draft preparation, WZ and S Z; writing—editing, ZW; supervision, ZW; project administration, WZ and ZW; funding acquisition, ZW. All authors have read and agreed to the published version of the manuscript.

## Funding

This work was supported by the National Natural Science Foundation of China (No. 81601692) and Technology Research from the Department of Education of Liaoning Province (No. JCZR2020013), and 345 Talent Project of Shengjing Hospital of China Medical University.

## Conflict of interest

The authors declare that the research was conducted in the absence of any commercial or financial relationships that could be construed as a potential conflict of interest.

## Publisher’s note

All claims expressed in this article are solely those of the authors and do not necessarily represent those of their affiliated organizations, or those of the publisher, the editors and the reviewers. Any product that may be evaluated in this article, or claim that may be made by its manufacturer, is not guaranteed or endorsed by the publisher.
