# Supplementary material for: Prognostic value of 12 m7G methylation-related miRNA markers and their correlation with immune infiltration in breast cancer
Source: Front Oncol. 2022 Aug 5;12:929363. doi: 10.3389/fonc.2022.929363 (PMC9389359; doi:10.3389/fonc.2022.929363)
Supplement: Supplementary file 1 [file Table_1.docx]

| id | HR | HR.95L | HR.95H | pvalue |
| --- | --- | --- | --- | --- |
| hsa-miR-3662 | 1.362126 | 1.178424 | 1.574464 | 2.90E-05 |
| hsa-miR-2115-5p | 1.161774 | 1.071206 | 1.259999 | 0.000293 |
| hsa-miR-483-3p | 1.001545 | 1.000651 | 1.002439 | 0.0007 |
| hsa-miR-21-3p | 1.000154 | 1.000064 | 1.000245 | 0.000861 |
| hsa-miR-6844 | 1.585681 | 1.187567 | 2.117257 | 0.001776 |
| hsa-miR-483-5p | 1.011606 | 1.004275 | 1.01899 | 0.001874 |
| hsa-miR-340-5p | 1.006342 | 1.002014 | 1.01069 | 0.004044 |
| hsa-miR-4675 | 1.192663 | 1.042627 | 1.364288 | 0.010214 |
| hsa-miR-3187-3p | 1.433034 | 1.08156 | 1.898726 | 0.012208 |
| hsa-miR-454-5p | 1.211689 | 1.039232 | 1.412765 | 0.014237 |
| hsa-miR-877-5p | 1.104448 | 1.010683 | 1.206911 | 0.028183 |
| hsa-miR-3150b-3p | 1.022771 | 1.00142 | 1.044577 | 0.03646 |
| hsa-miR-4501 | 1.087078 | 1.003964 | 1.177074 | 0.039643 |
| hsa-miR-629-3p | 0.945154 | 0.894961 | 0.998162 | 0.042762 |
| hsa-miR-556-3p | 0.667185 | 0.449974 | 0.989249 | 0.044035 |
| hsa-miR-200c-3p | 0.999963 | 0.999927 | 1 | 0.047479 |

Supplementary Table S1. 201 miRNAs related to the m7G methyltransferase target gene RNMT/FAM103A1 with differences
