# Supplementary material for: Prognostic value of 12 m7G methylation-related miRNA markers and their correlation with immune infiltration in breast cancer
Source: Front Oncol. 2022 Aug 5;12:929363. doi: 10.3389/fonc.2022.929363 (PMC9389359; doi:10.3389/fonc.2022.929363)
Supplement: Supplementary file 2 [file Table_2.docx]

| **id** |  | **Primers sequences** | **Tm** |
| --- | --- | --- | --- |
| hsa-miR-21-3p | forward | ccaacaccagtcgatgggctt | 59.7 |
| hsa-miR-200c-3p | forward | taatactgccgggtaatgatgga | 58.7 |
| hsa-miR-629-3p | forward | gttctcccaacgtaagcccagc | 60.4 |
| hsa-miR-340-5p | forward | ggcccttataaagcaatgagactgatt | 57.7 |
| hsa-miR-4501 | forward | ccgtatgtgacctcggatgaatca | 58.7 |
| hsa-miR-877-5p | forward | gtagaggagatggcgcaggg | 59.1 |
| hsa-miR-3662 | forward | ccgggaaaatgatgagtagtgactat | 56.7 |
| hsa-miR-4675 | forward | cggggctgtgattgaccagcag | 62.6 |
| hsa-miR-556-3p | forward | ccgcatattaccattagctcatcttt | 55.8 |
| hsa-miR-483-3p | forward | tcactcctctcctcccgtctt | 58.5 |
| hsa-miR-6844 | forward | cccggttctttgtttttaattcacag | 56.1 |
| hsa-miR-2115-5p | forward | ccgagcttccatgactcctgatga | 60.4 |

Supplementary Table S2. 12 miRNA Primers sequences used in our study
